# *KCNQ5* and *C9orf50* Methylation in Stool DNA for Early Detection of Colorectal Cancer

**DOI:** 10.3389/fonc.2020.621295

**Published:** 2021-01-29

**Authors:** Yaping Cao, Guodong Zhao, Mufa Yuan, Xiaoyu Liu, Yong Ma, Yang Cao, Bei Miao, Shuyan Zhao, Danning Li, Shangmin Xiong, Minxue Zheng, Sujuan Fei

**Affiliations:** ^1^ Department of Gastroenterology, Affiliated Hospital of Xuzhou Medical University, Xuzhou, China; ^2^ Institute of Digestive Diseases, Xuzhou Medical University, Xuzhou, China; ^3^ Zhejiang University Kunshan Biotechnology Laboratory, Zhejiang University Kunshan Innovation Institute, Kunshan, China; ^4^ State Key Laboratory of Bioelectronics, School of Biological Science and Medical Engineering, Southeast University, Nanjing, China; ^5^ Department of R&D, Suzhou VersaBio Technologies Co. Ltd., Kunshan, China; ^6^ Suzhou Institute of Biomedical Engineering and Technology, Chinese Academy of Sciences, Suzhou, China

**Keywords:** methylated *C9orf50*, methylated *KCNQ5*, stool DNA, colorectal cancer, early detection

## Abstract

**Background:**

Aberrant DNA methylation has emerged as a class of promising biomarkers for early colorectal cancer (CRC) detection, but the performance of methylated *C9orf50* and methylated *KCNQ5* in stool DNA has never been evaluated.

**Methods:**

Methylation specific quantitative PCR (qPCR) assays for methylated *C9orf50* and methylated *KCNQ5* were developed. The methylation levels of *C9orf50* and *KCNQ5* in 198 CRC patients, 20 advanced adenoma (AA) patients, 101 small polyp (SP) patients, and 141 no evidence of disease (NED) subjects were analyzed.

**Results:**

The methylation levels of both *KCNQ5* and *C9orf50* genes were significantly higher in CRC and AA groups than those in SP and NED groups, but showed no significant difference among different stages of CRC. The sensitivities of methylated *KCNQ5* and methylated *C9orf50* for CRC detection were 77.3% (95% CI: 70.7–82.8%) and 85.9% (95% CI: 80.0–90.2%) with specificities of 91.5% (95% CI: 85.3–95.3%) and 95.0% (95% CI: 89.7–97.8%), respectively. When *C9orf50* and methylated *KCNQ5* were combined, the clinical performance for CRC detection was similar to that of methylated *C9orf50* alone.

**Conclusions:**

Stool DNA based methylated *C9orf50* test has the potential to become an alternative approach for CRC screening and prevention.

## Introduction

Colorectal cancer (CRC) is one of the most widespread and lethal malignancies globally. It ranked the third in incidence and the second in mortality worldwide in 2018, accounting for more than 1.8 million new cases and over 0.86 million deaths (1). According to the same study, its rankings for China were the second for incidence and the fifth for mortality for the same year, leading to nearly 517,000 new cases and over 245,000 deaths. Moreover, due to an aging population and lifestyle changes toward a more Western diet and activity pattern in recent years, CRC incidence rate has steadily increased and the age of first diagnosis has decreased significantly in China (2).

As it is generally believed that it takes 10 years or more for adenomas, the precancerous lesions, to develop into sporadic CRCs (3), there is an ample time window to identify and treat CRC at its early stages to reduce incidence and mortality rates. Indeed, results from randomized controlled trials and observational studies have provided compelling evidence that early screening could lead to significant reduction of CRC incidence and mortality (4–6), leading to a multitude of national and international guidelines for early CRC screening (7).

Multiple CRC screening approaches have been used in the clinics including fecal immunochemical test (FIT), guaiac-based fecal occult blood test (gFOBT), colonoscopy, flexible sigmoidoscopy, and stool DNA test, which have their distinct advantages and disadvantages. For example, the low sensitivity for detecting stage I CRCs and advanced adenomas (AA) of annual FIT or gFOBT test has limited their effectiveness as screening tools for early stage CRC detection (8, 9). On the other hand, colonoscopy, the gold standard of CRC screening, has demonstrated higher sensitivities for the detection of precancerous lesions and early stage CRC than FIT and gFOBT tests (10–12). However, its significantly higher cost, invasiveness, complicated bowel preparation, and potential complications (13–15) have prevented its wide acceptance by Chinese population (16). Besides, it is hardly a primary CRC screening method in developing countries with limited medical resources and personnel such as China. In contrast, recent application of blood- and stool-based molecular diagnostic assays for early CRC screening in the clinics has demonstrated the feasibility of these alternative approaches. Cologuard, the first stool-based CRC screening test approved by the US Food and Drug Administration (FDA) with relatively high sensitivity and specificity, includes hemoglobin, multiple genetic mutations, and *BMP3* and *NDRG4* methylation sites as biomarkers (8). Similar to colonoscopy, its high cost and complex procedure renders it unfriendly to low- and moderate-income countries like China. Plasma-based Epi proColon 2.0 test, another FDA-approved CRC screening test (17), utilizes a single *SEPT9* methylation site as the biomarker. However, the sensitivities of *SEPT9* methylation were much lower than those of colonoscopy and Cologuard test, especially for precancerous lesions and early stage CRCs (18–20).

Recently, aberrant DNA methylation has emerged as a class of promising biomarkers for cancer diagnosis (21–23), and their successful clinical application for CRC screening and prevention has been demonstrated by Cologuard and Epi proColon 2.0 tests. In addition to *SEPT9*, *BMP3*, and *NDRG4* methylation biomarkers employed in the above two tests, aberrant methylation of other genes has been investigated as potential CRC biomarkers in the literature, including *SDC2* (24, 25), *SFRP1* (26–29), *SFRP2* (29–31), *GATA5* (32), *ITGA4* (33–36), *COL4A1* (33), *COL4A2* (33), *TLX2* (33), *VIM* (36–38), *cg10673833* (39), *GRIA4* (40), *VIPR2* (41), *EYA4* (42), *MAP3K14-AS1* (42), *MSC* (42), *CLIP4* (43), *C9orf50* (43), and *KCNQ5* (43). Some of these methylation biomarkers have been evaluated with both blood and stool samples for CRC detection but not methylated *C9orf50* and methylated *KCNQ5*. Whereas both methylated *C9orf50* and methylated *KCNQ5* exhibited good performance for early CRC detection with blood samples with sensitivities of 50.0 and 87.5%, respectively, for stage I CRC (43), their performance with stool samples was lacking. The aim of this study was to address this unanswered question and thus to evaluate them as potential biomarkers for a low-cost, convenient, and more accurate screening method urgently needed to promote early CRC screening in China.

## Materials and Methods

### Sample Enrollment and Collection

**Figure 1:**
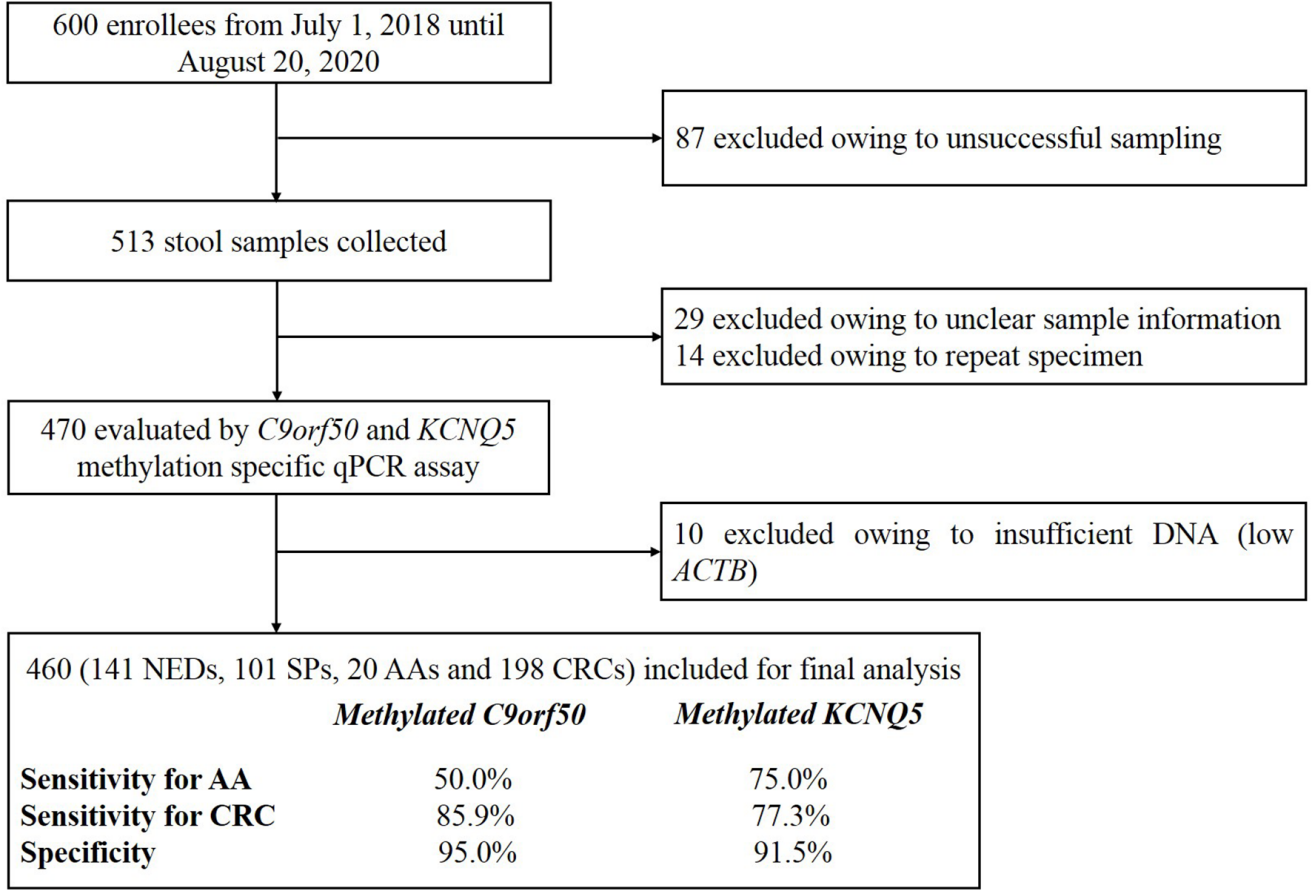
The flowchart of this study.

A total of 600 volunteers were recruited from July 1, 2018 until August 20, 2020 at the Affiliated Hospital of Xuzhou Medical University. All participants with colonoscopy results have acknowledged and signed the informed consent, and this study was performed according to the principles of the Helsinki Declaration and approved by the Institutional Review Board of the Affiliated Hospital of Xuzhou Medical University (Ethics Committee reference number: XYFY2020-KL122). Eighty-seven participants were excluded owing to unsuccessful sampling, resulting in 513 stool samples collected (**Figure 1**). Forty-three samples were subsequently excluded before quantitative PCR (qPCR) test due to missing sample information or repeated sampling. Finally, after excluding 10 samples due to low human gDNA amounts reflected by *ACTB* Ct values, 460 samples with valid qPCR results for next step analysis included 198 from CRC patients, 20 from advanced adenoma (AA, an adenoma measuring ≥ 10 mm in size, with high-grade dysplasia, or with ≥ 25% villous features) (44, 45) patients, 101 from small polyp (SP, an adenoma < 10 mm in size without high-grade dysplasia and villous histologic features, or hyperplastic polyp < 10 mm in size) patients, and 141 from no evidence of disease (NED, control subjects with normal colonoscopy results) subjects. Stool samples were collected 1–5 day before routine bowel preparation for colonoscopy with single-use disposable sampling boxes (46), and approximately 5 g stool samples were transferred to 50ml collection tubes containing 25 ml of preservative buffer with sampling spoons. All stool samples were stored at room temperature no more than 7 days before being transferred to −80°C for long-term preservation and storage.

### DNA Isolation and Methylation Testing

Stool human genomic DNA for each subject was isolated by VersaBio Human Genomic DNA FastPrep Kit (Suzhou VersaBio Technologies Co., Ltd., Kunshan, China) according to the previously published protocol (46). Briefly, the stool samples were homogenized at least for 1 min, and then centrifuged at 10,000 g for 20 min, and 150 μl supernatants were transferred into new 2 ml tubes for DNA extraction. Next, 500 μl preservative buffer was added to each supernatant and centrifuged at 20,000 g for 3 min. The resulting supernatant was then transferred to a new 2 ml tube, and 600 μl lysis buffer, and 20 μl proteinase K solution were added and subsequently incubated at 70°C for 10 min. Six hundred microliter ethanol was added into each sample and then loaded onto a spin column. After two washing steps, the stool human genomic DNA was eluted with 100 μl elution buffer. Bisulfite conversion of stool human genomic DNA and purification of the converted products followed the previously published procedure of a fast bisulfite conversion kit (Suzhou VersaBio Technologies Co., Ltd.) (46). One hundred and fifty microliter conversion buffer and 25 μl protection buffer were added to each purified DNA sample, and the resulting mixture was incubated at 80°C for 45 min. Next, 1 ml wash buffer A was added to each sample and loaded onto a spin column. After two washing steps, the converted DNA was eluted with 100μl elution buffer.

To analyze the methylation levels of *KCNQ5* and *C9orf50* genes in stool DNA, a triplex methylation specific qPCR (qMSP) assay based on Jensen S. et al. (43) was developed. All primers and probes for detecting methylated *KCNQ5*, methylated *C9orf50*, and internal control (*ACTB*) were synthesized by GENEWIZ BioTechnologies (Suzhou, China) and listed in **Supplementary Table 1**. VersaBio Multiplex Methylation Specific PCR Master Mix Kit (Suzhou VersaBio Technologies Co., Ltd.) was used. Thirty microliter reactions containing 15μl of bisulfite-converted stool DNA each were performed on ABI 7500 instrument (Applied Biosystems, Foster City, CA, USA) under the following conditions: initial activation at 95°C for 20 min, followed by 50 cycles at 95°C for 10 s, 56°C for 30 s and 72°C for 10 s, and a final cooling to 40°C for 30 s. Three PCR replicates were performed simultaneously for each sample.

### Data Analysis

*ACTB* Ct values were used to validate sample processing. The result for a stool sample was considered “invalid” if the *ACTB* mean Ct value of three qPCR reactions was greater than 41.0. The Ct values of methylated *KCNQ5* and methylated *C9orf50* were used to determine whether a stool sample was scored positive or negative for the corresponding methylation biomarker. The cutoff for methylated *KCNQ5* was all three qPCR reactions producing amplification signals (3/3 algorithm) with a mean Ct of less than 35.0. The cutoff for methylated *C9orf50* was all three qPCR reactions producing Ct values of less than 50.0 (3/3 algorithm). For two-biomarker combination of methylated *KCNQ5* and methylated *C9orf50*, a stool sample would be scored positive when either methylated *KCNQ5* or methylated *C9orf50* was scored positive. Mean Ct value of each sample in CRC, AA, SP and no evidence of disease (NED) groups was used to represent its target methylation level. Those reactions without amplification signals were set a Ct value of 50 (the maximal number of PCR cycles) for the mean Ct analysis (47). The receiver operating characteristic (ROC) curves were plotted to calculate the area under curve (AUC) values. All statistical analyses were performed with IBM SPSS for Windows Version 22.0. *Pearson* chi-square test was used for sensitivity comparison among groups at a significance level of *p* < 0.05, the differences in methylation levels have been analyzed using the Mann–Whitney U test.

## Results

A total of 513 stool samples were collected in this study, of which 43 were excluded due to insufficient sample information or repeated sampling. Four hundred and sixty samples returned valid data for methylated *KCNQ5* and methylated *C9orf50* qMSP assay. Among them, 198 were CRC patients, 20 were AA patients (**Supplementary Table 2**), 101 were patients with SPs, and 141 were NED subjects. For CRC group, 60.6% were male and the mean age was 61.7, including 5 stage 0, 32 stage I, 58 stage II, 59 stage III, 21 stage IV patients, and 23 patients of unknown stage (**Table 1**).

**Table 1:** Statistics of the participants in this study.

	All [N(%)]	Male [N(%)]	Female [N(%)]	Age (y) [median (range)]
**Number of participants**	460 (100.0)	268 (58.3)	192 (41.7)	55 (22–92)
**CRC**	198 (100.0)	120 (60.6)	78 (39.4)	63 (22–92)
Stage 0	5 (100.0)	4 (80.0)	1 (20.0)	67 (38–72)
Stage I	32 (100.0)	15 (46.9)	17 (53.1)	64 (30–86)
Stage II	58 (100.0)	41 (70.7)	17 (29.3)	63 (31–84)
Stage III	59 (100.0)	29 (49.2)	30 (50.8)	63 (38–84)
Stage IV	21 (100.0)	16 (76.2)	5 (23.8)	56 (41–78)
Unknown stage	23 (100.0)	15 (65.2)	8 (34.8)	68 (22–92)
**AA**	20 (100.0)	14 (70.0)	6 (30.0)	63 (46–76)
**SP**	101 (100.0)	62 (61.4)	39 (38.6)	55 (24–84)
**NED**	141 (100.0)	72 (51.1)	69 (48.9)	48 (22–83)

**Figure 2:**
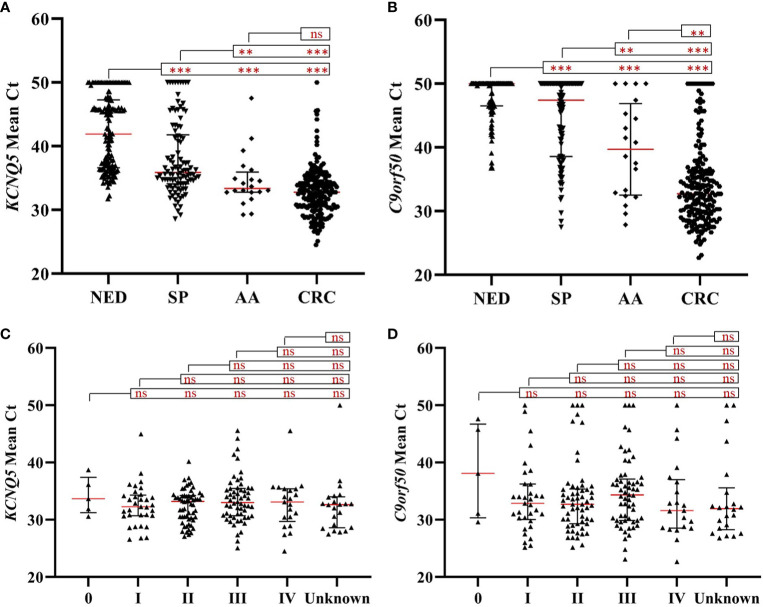
The methylation levels of *KCNQ5* and *C9orf50* in no evidence of disease (NED), small polyp (SP), advanced adenoma (AA), colorectal cancer (CRC) groups **(A, B)** and different stages of CRC **(C**, **D)**. **p < 0.01; ***p < 0.0001, ns, no significant difference according to Student’s *t*-test. Red lines represent the median methylation levels of *KCNQ5* or *C9orf50*. Mann–Whitney U test compared to methylation levels of *KCNQ5* and *C9orf50* in NED, SP, AA, CRC groups.

To determine whether the methylation levels of *KCNQ5* and *C9orf50* in stool DNA were capable of distinguishing CRC from other samples, the mean Ct values of each group was analyzed. As shown in **Figures 2A**, **B**, *KCNQ5* and *C9orf50* in CRC (*p*<0.0001), AA (*p*<0.0001) and SP (*p*<0.0001) groups all displayed significantly higher methylation levels than in NED group. The methylation levels of *KCNQ5* and *C9orf50* in CRC (*p*<0.0001) and AA (*p*<0.05 and *p*<0.01, respectively) groups were also significantly higher than those in SP group. *C9orf50* showed significantly higher methylation levels in CRC (*p*<0.01) than in AA group, but *KCNQ5* showed no significantly difference between CRC group and AA group (**Figure 2A**). In contrast, both *KCNQ5* and *C9orf50* showed no significant difference in the methylation levels among different stages of CRC (**Figures 2C, D** **)**.

**Figure 3:**
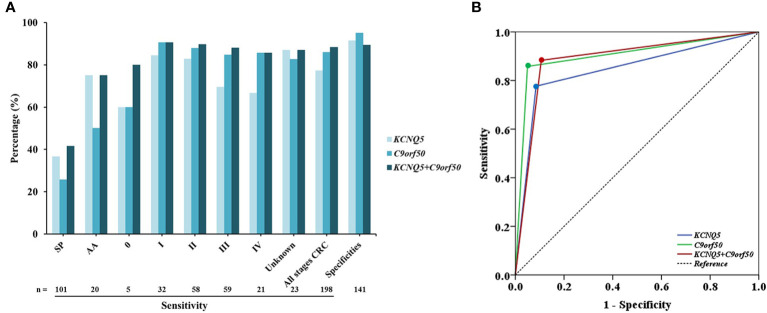
Sensitivities and specificities of methylated *KCNQ5* and methylated *C9orf50* in stool DNA for small polyp (SP), advanced adenoma (AA), and colorectal cancer (CRC) detection **(A)**, and the receiver operating characteristic (ROC) curves of methylated *KCNQ5* and methylated *C9orf50* in stool DNA for CRC detection **(B)**.

Methylated *KCNQ5* was positive in 36.6% (37/101) of SP, 75.0% (15/20) of AA, 60.0% (3/5) of stage 0 CRC, 84.4% (27/32) of stage I CRC, 82.8% (48/58) of stage II CRC, 69.5% (41/59) of stage III CRC, 66.7% (14/21) of stage IV CRC, and 87.0% (20/23) of CRC of unknown stage. The sensitivity and the specificity of methylated *KCNQ5* for detecting all stage CRC were 77.3% (95% CI: 70.7–82.8%) and 91.5% (95% CI: 85.3–95.3%). Methylated *C9orf50* was positive in 25.7% (26/101) of SP, 50.0% (10/20) of AA, 60.0% (3/5) of stage 0 CRC, 90.6% (29/32) of stage I CRC, 87.9% (51/58) of stage II CRC, 84.7% (50/59) of stage III CRC, 85.7% (18/21) of stage IV CRC, and 82.6% (19/23) of CRC of unknown stage. The sensitivity and the specificity of methylated *C9orf50* for detecting all stage CRC were 85.9% (95% CI: 80.0–90.2%) and 95.0% (95% CI: 89.7–97.8%). When methylated *KCNQ5* and methylated *C9orf50* were combined for CRC detection, the sensitivity and the specificity were 88.4% (95% CI: 82.9–92.3%) and 89.4% (95% CI: 82.8–93.7%), respectively (**Figure 3A**).

ROC analysis demonstrated the ability of methylated *KCNQ5* alone, methylated *C9orf50* alone, and the combination of methylated *KCNQ5* and methylated *C9orf50* to discriminate CRC from NEDs with AUC values of 0.846 (95% CI: 0.802–0.890), 0.904 (95% CI: 0.869–0.940), and 0.888 (95% CI: 0.849–0.928), respectively (**Figure 3B**). Sensitivities of methylated *KCNQ5* and methylated *C9orf50* in stool DNA for detecting CRC of different characteristics are summarized in **Table 2**. Methylated *KCNQ5* showed no significant difference of sensitivities among different genders, age groups, stages, tumor locations, tumor sizes, and differentiation statuses, but sensitivities of methylated *C9orf50* for detecting CRC of different locations showed statistically significant differences (*p*<0.05). Similar analysis was also performed on the 20 AA cases. As shown in **Supplementary Table 3**, both methylated *KCNQ5* and methylated *C9orf50* showed no significant sensitivity differences between different genders, polyp locations, and differentiation statuses. However, methylated *C9orf50* but not methylated *KCNQ5* exhibited significant sensitivity difference between different polyp sizes (*p*<0.05).

**Table 2:** Sensitivities of methylated *KCNQ5* and methylated *C9orf50* in stool DNA for detecting colorectal cancer (CRC) in different genders, age groups, stages, tumor locations, tumor sizes, and differentiation statuses.

	*KCNQ5*		*C9orf50*		*KCNQ5 + C9orf50*	
	Sensitivity (%)	*p-value*	Sensitivity (%)	*p-value*	Sensitivity (%)	*p-value*
**Gender**						
Male (n=120)	80.0	0.256	86.7	0.686	89.2	0.670
Female (n=78)	73.1		84.6		87.2	
**Age**						
<40 (n=10)	90.0	0.132	80.0	0.981	90.0	0.750
40–49 (n=20)	80.0		85.0		85.0	
50–59 (n=54)	66.7		85.2		85.2	
60–69 (n=57)	77.2		86.0		87.7	
70–79 (n=44)	79.5		86.4		90.9	
≥80 (n=13)	100.0		92.3		100.0	
**T stage**						
Tis (n=5)	60.0	0.706	60.0	0.271	80.0	0.298
T1 (n=5)	80.0		80.0		80.0	
T2 (n=41)	75.6		85.4		85.4	
T3 (n=89)	79.8		89.9		93.3	
T4 (n=25)	68.0		80.0		80.0	
N/A (n=33)	81.8		84.8		87.9	
**N stage**						
N0 (n=100)	81.0	0.053	87.0	0.522	89.0	0.322
N1 (n=43)	62.8		81.4		83.7	
N2 (n=22)	81.8		90.9		95.5	
N/A (n=33)	81.8		84.8		87.9	
**Location**						
Colon (n=78)	70.5	0.109	76.9	0.006	80.8	0.011
Rectum (n=113)	80.5		91.2		92.9	
N/A (n=7)	100.0		100.0		100.0	
**Size**						
≤4 cm (n=89)	76.4	0.952	86.5	0.828	88.8	0.879
>4 cm (n=75)	76.0		85.3		88.0	
N/A (n=34)	82.4		85.3		88.2	
**Differentiation**						
Low (n=25)	76.0	0.785	92.0	0.655	92.0	0.542
Moderate (n=100)	79.0		86.0		89.0	
High (n=29)	72.4		82.8		82.8	
N/A (n=44)	77.3		84.1		88.6	

p-values were calculated by Pearson chi-square test. N/A group data were not included for chi-square test.

## Discussion

Colorectal cancer is one of the most prevalent and lethal malignancies globally and in China. The best approach to reduce its burden on individuals and societies is through prevention by early CRC screening, which has been shown to reduce CRC incidence and mortality rates (48). Current methods for early CRC screening all have disadvantages for developing countries including China, such as relatively poor diagnostic accuracy for low-cost FIT (8) and gFOBT tests (9) and high demand on limited medical resources and personnel for diagnostically more accurate colonoscopy, sigmoidoscopy, and stool-based Cologuard test (49). Therefore, development of low-cost, convenient, and more accurate early CRC screening methods will help promote widespread acceptance of early CRC screening by the population especially in developing countries.

In this study, we have examined the clinical performance of stool-based methylated *C9orf50* and methylated *KCNQ5* tests for CRC and precancerous lesion detection (**Figure 3** and **Table 3**). Overall, the clinical performance of both methylation biomarkers for CRC detection in stool-based assays was similar to that in plasma-based assays (43) with two exceptions (**Table 3**). Stool methylated *C9orf50* test showed a much higher sensitivity for stage I CRC than plasma-based test (90.6 *vs*. 50.0%). On the contrary, plasma methylated *KCNQ5* test showed a much higher sensitivity for stage IV CRC than stool-based test (100.0 *vs*. 66.7%). One possible explanation could account for these differences between the results of stool test and plasma test (43). Stool DNA originated directly from cancer or polyp tissues in colon or rectum. However, after entering the blood stream, circulating tumor DNA were diluted by a person’s entire blood supply. Therefore, it is conceivable that tumor DNA was more concentrated in stool than in plasma, resulting in a higher sensitivity for CRC detection with stool samples. Such a phenomenon was also observed for methylated *SEPT9* and methylated *SDC2* in our previous studies (50, 56, 57).

**Table 3:** Comparison of clinical performance of single methylation biomarkers for colorectal cancer (CRC) and precancerous lesion detection from representative studies.

Sample type	Methylation biomarker		Sensitivity								Specificity	Reference
		SP	AA	Stage 0 CRC	Stage I CRC	Stage II CRC	Stage III CRC	Stage IV CRC	Stage 0 to II	All stage CRC		
Blood	*C9orf50*				50.0% (8/16)	79.4% (54/68)	81.8% (18/22)	85.7% (6/7)	73.8% (62/84)	76.1% (86/113)	90.8% (79/87)	(43)
	*KCNQ5*				87.5% (14/16)	83.8% (57/68)	72.7% (16/22)	100.0% (7/7)	84.5% (71/84)	83.2% (94/113)	95.4% (83/87)
Stool	*C9orf50*	25.7% (26/101)	50.0% (10/20)	60.0% (3/5)	90.6% (29/32)	87.9% (51/58)	84.7% (50/59)	85.7% (18/21)	87.4% (83/95)	85.9% (170/198)	95.0% (134/141)	This study
	*KCNQ5*	36.6% (37/101)	75.0% (15/20)	60.0% (3/5)	84.4% (27/32)	82.8% (48/58)	69.5% (41/59)	66.7% (14/21)	82.1% (78/95)	77.3% (153/198)	91.5% (129/141)
	*C9orf50* and *KCNQ5* combined	41.6% (42/101)	75.0% (15/20)	80.0% (4/5)	90.6% (29/32)	89.7% (52/58)	88.1% (52/59)	85.7% (18/21)	89.5% (85/95)	88.4% (175/198)	89.4% (126/141)
	*SEPT9*		66.7% (8/12)		86.7% (13/15)	94.4% (17/18)	78.3% (18/23)	77.8% (7/9)	90.1% (30/33)	83.3% (60/72)	92.1% (70/76)	(50)
	SEPT9*		50.0% (6/12)	100.0% (1/1)	63.6% (7/11)	93.3% (14/15)	77.8% (14/18)	75.0% (3/4)	81.5% (22/27)	79.8% (75/94)	96.0% (119/124)	(46)
	SDC2*		50.0% (6/12)	100.0% (1/1)	72.7% (8/11)	100.0% (15/15)	83.3% (15/18)	50.0% (2/4)	88.9% (24/27)	85.1% (80/94)	95.1% (118/124)
	*SDC2*		66.7% (2/3)	100.0% (3/3)	85.5% (47/55)	91.4% (64/70)	89.6% (86/96)	100.0% (21/21)	89.1% (114/128)	90.2% (221/245)	90.2% (221/245)	(25)
	*SFRP2*		61.8% (21/34)							87.0% (60/69)	93.3% (28/30)	(31)
	*NDRG4*									76.2% (66/87)	89.2% (14/16)	(51)
	*COL4A1*		58.4% (45/77)							88.8% (71/80)	88.0% (73/83)	(33)
	*COL4A2*		49.4% (38/77)							92.5% (74/80)	91.6% (76/83)
	*TLX2*		54.5% (42/77)							88.8% (71/80)	96.4% (80/83)
	*ITGA4*		41.6% (32/77)							82.5% (66/80)	96.4% (80/83)
	*SPG20*									80.2% (77/96)	100.0% (30/30)	(52)
	*FBN1*				91.7% (11/12)	63.3% (19/30)	73.3% (22/30)	66.7% (2/3)	71.4% (30/42)	72.0% (54/75)	93.3% (28/30)	(53)
	*GATA5*									83.9% (47/56)	82.5% (33/40)	(32)
	*p33ING1b*		62.9% (17/27)							73.8% (45/61)	95.0% (19/20)	(54)
	*HPP1*	36.8% (7/19)	70.0% (7/10)							71.2% (37/52)	100.0% (24/24)	(55)
	*SFRP1*	43.8% (14/32)	73.7% (14/19)							89.7% (35/39)	90.0% (18/20)	(27)
	*VIM*									72.5% (29/40)	86.9% (106/122)	(38)
	Cologuard**	17.2% (498/2893)	42.4% (321/757)		89.7% (26/29)	100.0% (21/21)	90.0% (9/10)	75.0% (3/4)	94.0% (47/50)	92.3% (60/65)	89.8% (4002/4457)	(8)

*Combined results from both training set and validation set. **The results of stool-based multi-target Cologuard test serve as reference. The biomarkers of Cologuard test include BMP3 and NDRG4 methylation, 7 KRAS mutations, and hemoglobin protein.

When compared to other methylation biomarkers in stool-based tests, the sensitivities of methylated *C9orf50* and methylated *KCNQ5* for all stage CRC detection, 85.9 and 77.3% respectively, fell within the range of other markers from 71.2% for methylated *HPP1* (55) to 92.5% for methylated *COL4A2* (33). So did their specificities of 95.0% for methylated *C9orf50* and 91.5% for methylated *KCNQ5* in NED or healthy controls, when compared to the range observed for other markers from 82.5% for methylated *GATA5* (32) to 100.0% for methylated *SPG20* (52). As more detailed evaluations of the clinical performance for detecting different stages of CRC were not reported for most of the other markers, the sensitivities of methylated *C9orf50* and methylated *KCNQ5* for early stage CRC (stage 0 to II) could only be compared to those of methylated *SEPT9* and methylated *SDC2*, which were very similar among all four methylation markers, 87.4% for methylated *C9orf50*, 82.1% for methylated *KCNQ5*, 90.1 and 81.5% for methylated *SEPT9* (46, 50), and 88.9 and 89.1% for methylated *SDC2* (25, 46). On the other hand, the sensitivities of most of the other methylation biomarkers for AA detection, from 41.6% for methylated *ITGA4* (33) to 73.7% for methylated *SFRP1* (27), were on par with those of methylated *C9orf50* and methylated *KCNQ5*, 50.0 and 75.0% respectively.

When compared to the FDA-approved multi-target Cologuard test (8), the clinical performance of methylated *C9orf50*, 85.9% sensitivity for all stage CRC with 95.0% specificity, appeared to be similar to that of Cologuard, 92.3% sensitivity for all stage CRC with 89.8% specificity, whereas 77.3% sensitivity for all stage CRC with 91.5% specificity of methylated *KCNQ5* seemed to be inferior to those of Cologuard. As far as precancerous lesions were concerned, the sensitivities of methylated *C9orf50* for AA and SP, 50.0 and 25.7% respectively, were similar to those of Cologuard, 42.2 and 17.2%. In contrast, the sensitivities of methylated *KCNQ5* for AA and SP, 75.0 and 36.6%, appeared significantly higher than those of Cologuard, suggesting a better performance in a screening setting where identification of precancerous lesions and early stage CRC was preferred.

Furthermore, when methylated *C9orf50* and methylated *KCNQ5* were combined, the clinical performance for CRC detection was similar to that of methylated *C9orf50* alone, 88.4 *vs*. 85.9% for sensitivity and 89.4 *vs*. 95.0% for specificity. Thus, the performance improvement of two-marker combination appeared to be minimal, particularly for precancerous lesions and early stage CRC detection.

Whereas this study was the first to examine the clinical performance of stool-based methylated *C9orf50* and methylated *KCNQ5* tests for early CRC detection, it did have a few limitations. For example, the number of AA samples examined was relatively small, and the mean age of NED group was younger than CRC patients. Thus increasing the number of enrolled AA patients and comparable participant distribution in all groups should be considered in future studies. Meanwhile, stool methylated *C9orf50* or methylated *KCNQ5* test has not been directly compared with previous published methylation based CRC screening methods, such as plasma methylated *SEPT9* test or Cologuard. Therefore, more validation studies in multiple clinical centers as well as a large prospective comparison study between different methods should be carried out in the future.

## Conclusion

In the present study, we demonstrated that stool-based methylated *C9orf50* test exhibited high sensitivities and specificity for the detection of precancerous lesions and all stage CRCs. Whereas stool-based methylated *KCNQ5* test also demonstrated high sensitivities for the detection of precancerous lesions and early stage CRCs, its sensitivities for late stage CRCs were markedly lower than those of methylated *C9orf50*. These results suggested that stool-based methylated *C9orf50* test has the potential to become an alternative approach for early diagnosis of CRC.

## Data Availability Statement

The original contributions presented in the study are included in the article/**Supplementary Material**. Further inquiries can be directed to the corresponding authors.

## Ethics Statement

The studies involving human participants were reviewed and approved by the Institutional Review Board of the Affiliated Hospital of Xuzhou Medical University (Ethics Committee reference number: XYFY2020-KL122). All participants have acknowledged and signed the informed consent, and this study was performed according to the principles of the Helsinki Declaration and approved by the institutional review board of the Affiliated Hospital of Xuzhou Medical University (Ethics Committee reference number: XYFY2020-KL122). The patients/participants provided their written informed consent to participate in this study.

## Author Contributions

YapC, GZ, MY, and YM performed the statistical analyses and drafted the manuscript. YanC, MY, XL, YanC, BM, SZ, and DL participated in sample collection and data analysis. GZ, SX, MZ, and SF conceived of the study and participated in the design and coordination of the study. All authors contributed to the article and approved the submitted version.

## Funding

This work was supported by grants from the Suzhou Technology Entrepreneur Angel Project (grant CYTS2018051), Key Technologies R & D Program for Social Development of Jiangsu Province (grant BE2019688), Kunshan Leading Talent Project (grant 00311), Suzhou Innovation and Entrepreneurship Leading Talent Program (grant ZXL2020046), and Key Technologies R & D Program for Social Development of Xuzhou (grant KC17184).

## Conflict of Interest

Authors GZ and SX were employed by Suzhou VersaBio Technologies Co. Ltd.

The remaining authors declare that the research was conducted in the absence of any commercial or financial relationships that could be construed as a potential conflict of interest.

## Supplementary Material

Supplementary Table 1 Primers and probes used in this study.
